# Pilot trial of Stop Delirium! (PiTStop) - a complex intervention to prevent delirium in care homes for older people: study protocol for a cluster randomised controlled trial

**DOI:** 10.1186/1745-6215-15-47

**Published:** 2014-02-05

**Authors:** Anne Heaven, Francine Cheater, Andrew Clegg, Michelle Collinson, Amanda Farrin, Anne Forster, Mary Godfrey, Liz Graham, Anne Grice, Rachel Holt, Claire Hulme, Ernie Lloyd, David Meads, Chris North, John Young, Najma Siddiqi

**Affiliations:** 1 Bradford District Care Trust (R&D Department), Lynfield Mount Hospital, Heights Lane, Bradford, West Yorkshire BD9 6DP, UK; 2 School of Nursing Sciences, Faculty of Medicine and Health Sciences, University of East Anglia, Norwich Research Park, Norwich NR4 7TJ, USA; 3 Leeds Institute of Health Sciences, Charles Thackrah Building, University of Leeds, 101 Clarendon Road, Leeds LS2 9LJ, UK; 4 Clinical Trials Research Unit, Leeds Institute of Clinical Trials Research, University of Leeds, Leeds LS2 9JT, UK; 5 Department of Elderly Medicine, Pinderfields Hospital, Mid Yorkshire NHS Trust, Pinderfields Hospital, Aberford Road, Wakefield, West Yorkshire WF1 4DG, UK; 6 Bradford District Care Trust, Meridian House, Bradford Road, Keighley BD21 4AD, UK

**Keywords:** Pilot, Feasibility, RCT, Complex intervention, Older people, Delirium, Care home

## Abstract

**Background:**

Delirium (or acute confusion) is a serious illness common in older people, in which a person’s thinking and perceptions may be affected. Reducing delirium is important because of the considerable distress it causes, and the poor outcomes associated with it, such as increased admissions to hospital, falls, mortality and costs to the National Health Service (NHS). Preventing delirium is possible using multicomponent interventions; successful interventions in hospitals have reduced it by one-third. However, there is little research to guide practice in care homes, where it is common because of the clustering of known risk factors (older age, frailty, and dementia). In previous work we developed a multicomponent intervention to prevent delirium in care homes, called Stop Delirium! The intervention was based upon evidence from the research literature relating to the prevention of delirium and on strategies to change professional practice. Before starting a large costly trial of Stop Delirium!, this pilot study will test and help improve the design and feasibility of the trial protocol.

**Methods/Design:**

We plan to conduct a cluster randomised pilot trial in 14 care homes (independent residential and nursing). Following recruitment of residents (over 60 years, consenting or with consultee agreement, able to communicate in English, and not in palliative care) participating homes will be randomised, stratified by size of home and proportion of residents with dementia. Stop Delirium! will be delivered to intervention homes over 16 months, with controls receiving usual care. The primary outcome measure will be the presence of delirium on any day during a one-month post-intervention period.

We will collect data to determine 1) recruitment and attrition rates, 2) feasibility of various outcomes measurements, and 3) feasibility of capturing health resource use (resident diaries and by examining health records). We will estimate the between-cluster variation for the primary outcome, delirium occurrence.

**Discussion:**

This pilot study will refine methods for the definitive trial. The lessons learnt will also contribute to implementing National Institute for Health and Clinical Excellence (NICE) delirium guidelines, which recommend multicomponent interventions for delirium prevention.

**Trial registration:**

ISRCTN27972532.

## Background

Delirium is a distressing but preventable condition that is common in older people. It is associated with increased morbidity, mortality, functional decline, hospitalisation, and significant healthcare costs [1,2]. Despite its high prevalence and poor outcomes, delirium has been under-researched and neglected in clinical practice [1,3]. Most research on delirium has focused on hospital patients. Another (expanding [4]) high risk group is residents of care homes for older people. The burden of delirium is likely to be considerable in residents of long-term care given the clustering of known delirium risk factors [5], especially the high prevalence of dementia. Individuals admitted to hospital from long-term care have higher rates of delirium [6], and institutional residence has been identified as a risk factor for post-operative delirium [7]. However, there have been few good-quality studies, investigating delirium in long-term care. A systematic review [8] found no studies of delirium incidence and no studies from the United Kingdom (UK). The median point prevalence of delirium was reported to be 14% in studies reflecting the UK model of long-term care [8].

We know that delirium can be prevented (reduced by one-third) in hospitals [9-11]. Successful interventions are multifaceted and rely on a combination of staff education and systematic screening for modifiable risk factors (attending to dehydration, pain, sensory impairments and poor mobility). These are areas of care that should be equally applicable to long-term care settings. It is not yet known, however, whether a similar approach might be effective in care homes. The recent National Institute for Clinical Excellence (NICE) delirium guideline recommends that such interventions should be implemented in long-term care given the likely benefits. In addition, it recommends that the clinical and cost effectiveness of these interventions should be investigated as a research priority [12].

A further reason to focus on delirium is its link to care quality [13], which has been a subject of concern for some time in long-term care [14,15]; training of staff has not kept pace with the changing demands of care homes [16,17], and there is poorer management of medical conditions, increasing the need for unplanned hospital admissions and general practitioner (GP) consultations [18]. Achieving improvements in care quality is challenging [19]. Optimum delirium care is fundamentally the provision of basic good quality supportive care [13]. For staff inured to endless messages to improve quality, a focus on delirium prevention may be the ‘Trojan horse’ through which this can be achieved [20]. There are additional potential benefits: reducing morbidity, hospital admissions and healthcare costs.

In previous work, we used the Medical Research Council (MRC) framework for the development of complex interventions [21] to design the ‘Stop Delirium!’ [22] intervention to prevent delirium in care homes.

The Stop Delirium! intervention - essentially an enhanced educational package for care home staff - incorporates strategies to change practice, such as adapting to the local context, interactive teaching methods, promoting ownership and championing. This approach to delirium prevention is based upon and supported by the research literature from hospital settings and is consistent with NICE guidelines.

In preparation for this pilot trial, we have developed a standardised Stop Delirium! toolkit including a manual for the Delirium Practitioner and educational resources for care home staff.

**Figure 1:**
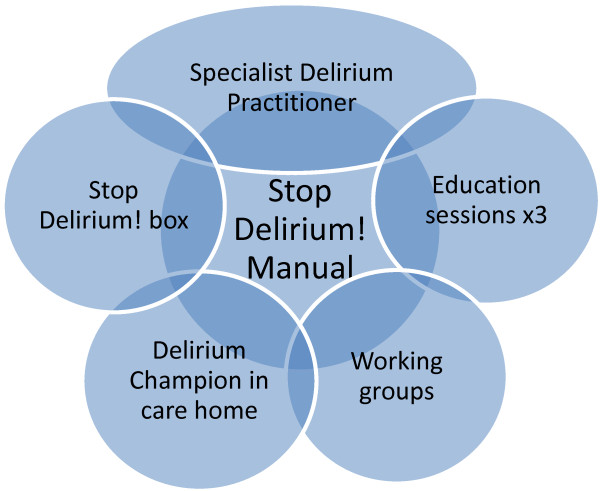
The individual components of Stop Delirium!

**Figure 2:**
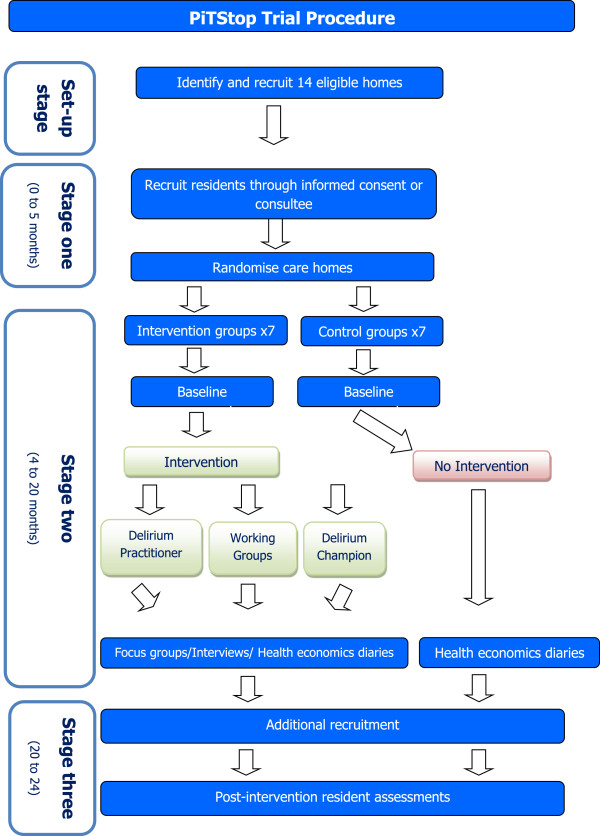
Flowchart of the PiTStop trial procedure.

Each of the intervention elements is discrete but interlinked. The individual components of Stop Delirium! are shown in Figure 1. We have now initiated a pilot trial as the first stage in an evaluation programme for this complex intervention. A flowchart of the trial procedure is shown in Figure 2.

### Aims and objectives

The primary hypothesis for the main trial will be that Stop Delirium! is a cost-effective way to prevent delirium episodes in residents of older people’s care homes. The secondary hypothesis will be that this will lead to a reduction in admissions to hospital from this population. The main objective of this pilot trial is to gather process, management, resource and scientific data to inform the design of a definitive trial. The principle objectives of this pilot and the corresponding research questions are outlined in Table 1.

**Table 1:** Principle objectives, research questions and outcomes

**Principle objectives**	**Research questions**	**Outcomes**
**Process:** To provide an estimate for the number of care homes needed for the main trial	What are the potential recruitment and attrition rates for enrolment of residents?	
**Resources:** To investigate the acceptability of the proposed measurements to residents and the most appropriate informant	Which type of data collection diaries (care home resident level), designed by the team to capture health and social care resource use, are most acceptable to residents and staff (>75% completed)? And are these diaries valid (contain information that can be used to calculate costs)?	Activities of daily living Number of falls during previous 6 months
		Mortality during previous 6 months
		Hospital admissions during previous 6 months including total length of stay; number of admissions; time to first admission
		Health-related quality of life
	Which health-related quality of life measure, the EuroQol (EQ-5D 3 L) [23], ASCOT Social Care-Related Quality of Life (SCRQoL) [24] or the DEMQOL-V4 [25] is more acceptable to residents (greater % of questionnaires completed)? And are resident and proxy reports of HRQoL comparable?	
	Are baseline and outcomes measures outcome rates adequate (achieving >75% complete data for each)? And what resources are required in determining these outcomes?	
**Management:** To assess the adherence to, and sustainability of Stop Delirium!	What are the adherence rates for the various components of the intervention? And what are the facilitators and barriers for sustainability and integration into routine care, after the intervention?	Total number of medications Health related quality of life Health and social care costs as measured by health related quality of life measures and health and social care resource use
	What are the costs from the perspective of the service provider (health and social care) associated with delirium and with Stop Delirium?	
**Scientific:** To determine the most appropriate method to capture resource use in this setting and population and costs of delirium and of delivering the intervention	Can a high (>90%) coverage for delirium screening be achieved using the short Confusion Assessment Method (CAM) [26]? And can a high completion rate (>90% complete) be achieved for the Delirium Rating Scale-Revised (DRS-R-98) [27] in those positive for CAM? Is delirium be determined reliably (>90% inter-rater reliability) using these instruments?	Delirium severity: proportion of residents with severe delirium during a one-month period
		Delirium duration (days positive for delirium) during a one month period
		Delirium incidence on any on any day during a one-month post-intervention period
		Mortality during previous 6 months
		Hospital admissions during previous 6 months including total length of stay, number of admissions, time to first admission
	What are the rates of delirium and admission to hospital in residents in intervention homes compared to control homes post intervention? And which measure yields more complete hospital admission data: length of stay, number of admissions or time to admission?	
To estimate the rates of delirium and hospital admissions in intervention and control homes	What is the intraclass coefficient (ICC) for the proposed primary outcome, delirium occurrence?	The primary outcome for this study is the presence of delirium on any day during a one-month post intervention period determined by screening with short version CAM [26] on alternate days (except Sundays) and confirmed for those positive or borderline using the Delirium Rating Scale-98 (DRSR98) [27].

## Methods/Design

### Design

We plan a pilot cluster, randomised, controlled trial conducted in 14 care homes for older people over 24 months, an embedded health economic feasibility study, and individual and focus group interviews to investigate sustainability and integration of the intervention.

### Population

The study will be conducted within the boundaries of Bradford Metropolitan District Council, West Yorkshire, in the north of England.

#### Inclusion criteria for care homes

The inclusion criteria for care homes will be as follows:

1. Care homes for older people in Bradford (nursing and residential)

2. Run by an independent provider (private, voluntary or non-profit making)

3. Within catchment area for Bradford District Care Trust Older People’s Community Mental Health Teams

#### Exclusion criteria for care homes

The exclusion criteria for care homes will be as follows:

1. Local Authority homes - these are homes run by local government. The number of care homes for older people run by local authorities is decreasing and the money is being transferred to third party providers. Therefore, it is believed that the intervention is less likely to be implemented in local authority homes.

2. Specialist homes (those focusing on stroke rehabilitation, except for those specialising in providing dementia care)

3. Care homes involved in other projects likely to impact on study (initiatives to reduce hospital admissions)

#### Inclusion criteria for residents

All residents present in the care home at the time of baseline assessments will be considered for participation.

#### Exclusion criteria for residents

The exclusion criteria for residents will be as follows:

1. Unable to participate in assessments because of severe communication difficulties or severe dementia.

2. Receiving end-of-life care

3. Non-English speakers

Whilst we recognise that the exclusion of non-English speakers is restrictive, we feel that it is appropriate at this trial stage. The number of Black and Minority Ethnic elders in Bradford care homes is thought to be low and the validated assessment tools are not available in South Asian languages. Recruitment will be monitored to ascertain how many people are excluded for this reason to inform the inclusion criteria of a future trial.

### Sample size

For a definitive trial, in the absence of empirical data on which to base an estimate of effect size from care homes, we plan to extrapolate from results obtained in hospital studies [9,10]. This approach is reasonable given similarities in the population and processes of care. Although care home residents may be less physically unwell, limiting the scope for impact, this is balanced by increased opportunity for staff to benefit from training and improve practice [17,28] and for residents to receive greater benefit due to longer exposure staff practice changes than is the case for short admission spells in hospitals.

For an individually randomised trial, 809 participants would be required per group to provide 80% power at 5% significance level to detect a reduction of one-third in delirium rates (found in delirium prevention studies in hospital settings). The assumed control rate of delirium is 14% (the median-point prevalence of delirium found in our literature review [8]).

The attrition rate would reduce the number of participants with a valid outcome within a site (cluster) and therefore affects the cluster size. When designing a cluster randomised trial, the cluster size, together with the intraclass coefficient (ICC), is used to estimate the design effect, which increases the sample size (compared to an individually randomised trial) to account for the clustering of outcomes.

### Recruitment

#### Care home recruitment

Eligible care homes in the Bradford District will be identified from the Care Quality Commission (CQC) public listing and the managers invited to return an expression of interest (EOI) in the study by post. Homes returning a positive EOI and non-respondents will be followed up with a telephone call. Care home managers who confirm their interest or have further queries will be visited in person to explain the study objectives, timescales and involvement required.

#### Resident recruitment

Care home managers will be asked to identify eligible residents in their care home. For those clearly not eligible, the following non-identifiable data will be recorded: care home, age, sex, ethnicity, and reason for exclusion.

All eligible residents will be asked by care home staff if they are willing to be seen by researchers so they can explain the study. If they agree, a researcher, accompanied by a member of care home staff will then explain the study to the resident and assess for capacity to consent. If residents have capacity they will be asked for consent to participate in the study. Residents who consent will also be asked about their views on future participation should their capacity fluctuate (through worsening dementia or a delirious episode). For residents without capacity, the advice of a consultee will be sought if there is no advanced directive in place pertaining to participation in research.

A protocol amendment has been put in place in order to undertake further recruitment of residents at 12 months post-intervention (2 to 4 months prior to the outcomes assessments). This will mitigate the effect of attrition in this extremely frail and elderly population and subsequent loss to follow-up. It will also allow us to investigate the feasibility of alternative approaches to the timing of recruitment.

Additional recruitment will take place in all care homes participating in the study. Care will be taken to spread additional recruitment over both the intervention and control arms. Residents will only be considered for recruitment at this stage if they have moved into the home subsequent to the first round of recruitment (those who have already been approached and refused will not be approached again). All other eligibility criteria will apply.

Additional residents will be recruited during the intervention and will provide baseline and follow-up assessment data in the same way as those recruited earlier.

We are conscious that a two-tier recruitment strategy may lead to differential recruitment, as randomisation to the intervention or control arm has already taken place; however, additional recruitment will not be used as a covariate in analyses.

### Randomisation

Homes will be randomised on a 1:1 basis to receive either active intervention (Stop Delirium!) or control (usual care) by the Leeds Clinical Trials Research Unit (CTRU), stratified by care home size (<20 or under, ≥20 residents) and the percentage of residents with dementia (<62%, ≥62%). Randomisation will take place only after the baseline recruitment and assessment of residents have been completed for each home.

### Blinding

Due to the nature of the intervention, it will not be possible to blind participants or the research team to allocation. Outcome measures will be collected by researchers having no role in the intervention and having no explicit knowledge of the allocations.

### Intervention

#### Stop Delirium!

Intervention homes will receive the Stop Delirium! package. This is a multicomponent intervention designed to prevent delirium in care home residents, based on the best delirium prevention and practice change evidence. It aims to modify key resident and environmental delirium risk factors (pain, infections, dehydration, poor nutrition, constipation, polypharmacy, sensory impairment, limited mobility and sleep disturbance) by improving the quality of care. A specialist delirium practitioner (DP) leads a delirium prevention educational process such that care home staff feel empowered, develop relevant skills and are able to identify opportunities for delirium prevention through the development of local care pathways and solutions tailored to the home environment. Key components (tested and refined in our previous feasibility study [22]) include:

1. Three 20-minute small group interactive education sessions for all staff in the home delivered by the DP and then offered monthly to new staff.

2. Monthly Staff Working Groups facilitated by the DP to identify key priority areas for delirium prevention in the home. Participants are drawn from staff volunteers attending education sessions.

3. A Delirium Box containing educational and implementation materials is established in each home by the home manager, supplemented by bespoke materials produced by staff members of the care home Working Groups.

4. The identification of delirium champions by the DP, who are then trained to deliver the education sessions and to facilitate Staff Working Groups under supervision, with a view to sustaining the intervention.

5. A manualised intervention to provide a consistent educational experience and approach to developing the workshops. Specific features of the intervention can be found on the European Delirium Association website [29].

The intervention will be in place for 16 months. This is 6 months longer than was allowed for in the previous feasibility phase. This period is intended to provide sufficient time for any change in practice to embed and have an effect.

#### Control

Residents in control homes will receive ‘usual care’ during the study but care homes will be offered the intervention at study end.

### Outcomes

Outcomes aligned to the feasibility objectives and research questions are detailed in Table 1.

### Data collection

Data will be collected electronically at each of the 14 care home sites, using portable, password-protected, encrypted devices (tablets). Each tablet will be configured to ease the burden on residents by allowing pre-population of data fields (where information has already been gathered by another assessment tool) and to allow intermittent breaks in data collection if necessary. System checks will flag all missing data to the researcher in real time which will ensure that the data sets are as complete as possible.

For each home in the study the following information will be recorded at baseline:

#### Care home data

Data collected from the care home records will include:

1. Name of provider.

2. Type of care (nursing only or nursing and dementia).

3. Number of residents.

4. Number of staff, including skill mix.

5. Hospital admissions over the previous 6 months.

6. Falls over the previous 6 months.

7. Any current educational programmes or other relevant initiatives.

#### Resident data

Screening and baseline data for residents will be collected before randomisation. Post-intervention outcome data will be collected at 16 months post-randomisation.

### Screening

The following anonymised screening, information will be collected for all residents within the care home: gender, age, ethnicity and eligibility for inclusion. Reasons for non-eligibility will be recorded.

### Baseline assessments

For all residents recruited, the following information will be recorded through examining care home records or administering tests. Baseline assessment data will include:

1. Initials

2. NHS number and hospital ID

3. GP name and address

4. Age

5. Sex

6. Medications - total number and names

7. Co-morbidities (Charlson index [30])

8. Activities of Daily Living (ADL) [31]

9. Hearing Impairment

10. Visual impairment (Snellen test card at 3 meters, acuity below 6/18 assessed as visually impaired)

11. EuroQoL EQ-5D and EQ5D proxy [23]

12. Either the Social Care Related Quality of Life (SCR-QoL) [24] or Dementia Quality of Life DEMQOL and DEMQOL-Proxy [25]

13. Delirium

Delirium assessments will be undertaken using a three step process:

1. Assessment of cognitive impairment using the Six Item Cognitive Impairment Test (6-CIT) [32,33]

2. Short Confusion Assessment Method (CAM) [26]

3. In those screening positive on CAM, the Delirium Rating Scale Revised-98 (DRS-R-98) [27] (using a cut-off score of >17.75)

### Post-intervention assessments

Researchers, blind to allocation, will collect data 16 months from randomisation - deemed to be the start of the intervention - through administering tests on alternate days (except Sundays) for a period of one month and examining individual care home and hospital records. Researchers will record;

1. Presence of delirium on any day during a 1-month post-intervention period. Screening will use the 6-CIT and short version CAM. Presence or absence of delirium for those positive will be confirmed using the DRS-R-98 (as at study baseline). Ratings for a 10% subsample will be compared to determine inter-rater reliability.

2. Delirium severity (proportion of residents with DRS-R-98 severity scale score >15.25 at any assessment) during the 1-month post-intervention period.

3. Delirium duration (days positive for delirium) during the 1-month post-intervention period.

4. Number of medications.

5. Activity of Daily Living (ADL) scores.

6. Falls during previous 6 months.

7. Mortality during previous 6 months.

8. Hospital admissions during previous 6 month including;

a. total length of stay

b. number of admissions

c. time to first admission

The 6-CIT is a dementia screening tool used in primary care. The short CAM is a four-question instrument used extensively for delirium screening and diagnosis [26]. The DRS-R-98 is a valid measure of delirium occurrence and severity [27]. We know that the training of the researcher affects performance of these instruments [34]. Researchers will be trained before both the baseline and post-intervention assessments. As part of the analysis, we will also check inter-rater reliability at baseline by comparing ratings for a 10% subsample.

If a resident scores positive on the short CAM, indicating a possible delirium, subject to prior consent, the care home manager will be informed in writing on the day of assessment so that appropriate action can be taken. A copy of this letter will be held on file with the consent forms.

#### Acceptability and burden of assessments

Time taken for completion of assessments will be recorded contemporaneously. Rates of completion for all outcomes measures or reason for noncompletion will be recorded. In addition, note will be taken of who completes the health-related quality of life measures (residents or proxies (carer or relative)).

#### Treatment data

*Intervention compliance.* Adherence to the components of the intervention at 4, 8 and 16 months from randomisation - early middle and late phases of delivery - will be recorded using a standardised *pro-forma*. Data recorded will include degree of completion for each component (education sessions, working groups) and reasons for noncompliance, allowing calculation of adherence rates for each, and identification of components that may need alteration before use in a definitive trial.

*Concomitant interventions.* Any new initiatives or projects introduced during the study period by other agents (researchers, health organisations or local authorities-especially those aimed at reducing hospital admissions and falls - will be recorded at 4, 8 and 16 months from randomisation).

#### Outcomes for the economic evaluation

Health and social care use by residents will be recorded using specifically designed data collection forms/diaries. Data will be collected at 4, 8 and 16 months from randomisation. Contemporaneous study records (of salaries and travel expenses) will be kept to determine resource use in implementing the intervention. We will collect the bulk of resource use data from care home records but will test the accuracy of this method by comparing the results with those from a subset of individual resident care records and with centralised records of events, such as hospitalisation from the Health and Social Care Information Centre (HSCIC).

Unit financial costs for health and social care resources will be obtained from national sources (Personal Social Services Research (PSSRU) Unit Costs of Health and Social Care, British National Formulary (BNF) and NHS reference cost databases) and used to determine costs. Utility (HRQoL) values will be determined from either EQ5D-3 L, SCR QoL or DEMQOL-P-4D.

#### Interviews

We will investigate opportunities and barriers to sustainability for Stop Delirium! and its integration into routine practice through qualitative interviews with key staff. Focus groups will take place at the start and end of the intervention period. Two groups will comprise either nursing or care staff from across all intervention homes. Individual interviews with a manager and a Delirium Champion from each intervention home will take place 16 months from randomisation.

### Analysis

In this pilot trial, quantitative analysis will focus on descriptive statistics and will focus on confidence interval estimation rather than formal hypothesis testing. An intention-to-treat-analysis of clinical data will be undertaken blind to allocation. Any statistical analysis in a definitive trial would include multilevel modelling to account for the cluster design. Qualitative data from interviews and focus groups will be analysed combining a grounded theory (constant comparison) approach and narrative analysis to develop understanding of the factors (such as contextual, leadership, and organisational structures and knowledge, beliefs and behaviours of staff) that facilitate or inhibit sustainability of the intervention and its integration into routine practice over time.

#### Baseline assessments

A preliminary analysis consisting of a series of descriptive tables summarising baseline characteristics of a) the 14 care homes and b) residents in the study will be presented. Summary and comparison statistics will be used to describe and compare the intervention and control arms. The baseline delirium prevalence (residents positive for delirium/total number of residents screened) will be determined from the baseline data.

#### Outcome assessments

The delirium occurrence rate for intervention and for control arms will be calculated (number of residents with an episode of delirium in one month/total number of residents screened). A 95% confidence interval for the difference in rates will be constructed. Intraclass coefficients will be calculated for delirium occurrence using data from the post-intervention period. Summary statistics and comparisons between the intervention and control arms post-intervention will also be calculated for all primary and secondary outcomes as detailed previously. Recruitment and attrition will be summarised, together with rates of consent, consultee consent, withdrawals and deaths. In addition, Kaplan-Meier survival curves will be constructed for the following outcomes:

1. Time to first hospital admission in previous 6 months.

2. Mortality in previous 6 months.

Outcomes for hospital admission and mortality are assessed starting 10 months after randomisation. This is because we hypothesise that the intervention will not be able to influence outcomes before that time as it provides education for staff, which they need to operationalise into actions that will then affect residents. We have not therefore collected data for events occurring between randomisation and 10 months for hospital admission. We will have information on deaths and will present a summary of these data for the intervention and the control homes.

Data from the first and second stages of resident recruitment will be analysed together with a separate sensitivity analysis to investigate the impact of the later phase.

#### Data quality

The quality of the data will also be assessed using markers for completeness and reliability (completion rates of delirium screening and rating instruments and measures of inter-rater reliability). In addition, we will determine which variable yields more complete hospital data: length of stay, number of admissions or time to admission.

### Patient and public involvement

A number of patient and public involvement strategies have been used to inform the development and conduct of the study. The opinion of members of the Bradford Older People’s Forum was sought in developing the study proposal. Two care home managers from the feasibility phase along with a Care Home Quality Visitor and a relative of a care home resident are members of the Advisory Board, which has a monitoring role. Care home residents have also reviewed written and audio-visual material for use in recruitment of residents at the design stage. Lay representatives with specialist interest sit on the Implementation Team that steers the project and are involved in the production of information, raising awareness with residents, relatives and care home staff; they will also be involved in the facilitation of focus groups with care home staff.

Most of the lay representatives for the study are experienced and involved in other similar activities (representatives on other Boards and committees in health and social care). All lay representatives were given the study protocol and background reading on delirium before involvement and will be recompensed for their time in accordance with INVOLVE recommendations [35]. Care homes will receive a one-off payment of £400 in recognition of the staff input.

### Trial governance

A multidisciplinary Implementation Team will guide the operational management of the trial, with responsibility for overall supervision of the study on behalf of the trial sponsor (Bradford District Care Trust) and trial funder (NIHR). The team comprises lay representatives, statisticians, health economists, trial managers, consultants in older people’s care, and experts in complex interventions and qualitative studies. In addition, an independent Advisory Board, chaired by the National Clinical Director for Older People, and including two lay representatives, two care home managers (independent of sample), experts in older people’s care and care homes, will oversee the trial. The primary role of the Advisory Board is *‘to ensure that the rights, safety and well-being of the trial participants are the most important considerations, and prevail over the interests of science and society.’*

This trial is being conducted in accordance with the principles of Good Clinical Practice and the NHS Research Governance Framework and all data are held in accordance with the Data Protection Act and Caldicott principles.

The study protocol received ethical approval from the Yorkshire and Humber Research Ethics Committee on 24/01/2012 (ref: 12/YH/0018). Further approval was given for additional recruitment and a change of quality of life assessment tool on 11/02/2013.

## Discussion

We have made the following modifications to the original planned protocol: i) increased the number of homes to be included from 12 to 14; ii) included residential as well as nursing homes; iii) introduced additional recruitment of residents at 12 months post-randomisation of homes (2 to 4 months before the post-intervention outcomes assessments); and iv) replaced the SCRQoL with the DEMQOL at post-intervention data collection. These changes will help us investigate the feasibility of alternative approaches to recruitment and quality of life assessments. Modifications have been agreed on by the study sponsor and funder, and have received Ethics Committee approval.

Although yet to be confirmed by the results of this pilot trial, we anticipate that the definitive trial will have a shorter intervention period (between 10 to 16 months), that recruitment of residents will take place nearer to the outcome assessments, and that an alternative method of delirium screening will be used.

## Trial status

Recruitment is ongoing. The second stage of recruitment started in May 2013.

## Abbreviations

CAM: Confusion Assessment Method; CQC: Care Quality Commission; CTRU: Leeds Clinical Trials Research Unit; DP: delirium practitioner; DRS-R-98: Delirium Rating Scale Revised-98; EOI: expression of interest; GP: general practitioner; ICC: intraclass coefficient; ITT: intention-to treat analysis; MRC: Medical Research Council; NHS: National Health Service; NICE: National Institute for Health and Clinical Excellence; NIHR: National Institute for Health Research; RCT: randomised controlled trial; RfPB: research for patient benefit; 6-CIT: Six Item Cognitive Impairment Test.

## Competing interests

The authors declare that they have no competing interests.

## Authors’ contributions

AH has responsibility for the day-to-day management of the trial and is involved in data collection. She drafted the manuscript for comment. NS is the lead grant holder; she developed the intervention and led the protocol development. FC, AJF, AF, MG, CH and JY are co-applicants and contributed to the final protocol. Other authors had specialist input in the project: MC -statistical analysis; LG -trial management; DM -health economics; CN -community psychiatric liaison in care homes; AG and EL -lay representation; AC and RH -consultants in older people’s medicine. All authors are members of the project Implementation Team and have commented on draft manuscripts and approved the final version for publication.

## Authors’ information

Dr Siddiqi is a Consultant Psychiatrist at Bradford District Care Trust and Honorary Senior Lecturer at the Leeds Institute for Health Sciences.

## Registration

The study was registered as an International Standard Randomised Controlled Trial (ISRCTN27972532) on 17/05/2012. It is also registered with the United Kingdom Clinical Research Network.
